# Synovial Macrophage and Fibroblast Heterogeneity in Joint Homeostasis and Inflammation

**DOI:** 10.3389/fmed.2022.862161

**Published:** 2022-04-25

**Authors:** Katharina Knab, David Chambers, Gerhard Krönke

**Affiliations:** ^1^Department of Internal Medicine 3-Rheumatology and Immunology, Friedrich-Alexander University Erlangen-Nürnberg (FAU), Universitätsklinikum Erlangen, Erlangen, Germany; ^2^Deutsches Zentrum für Immuntherapie (DZI), Friedrich-Alexander University Erlangen-Nürnberg (FAU), Universitätsklinikum Erlangen, Erlangen, Germany

**Keywords:** macrophages, fibroblasts, rheumatoid arthritis, synovial tissue, single-cell sequencing, inflammation

## Abstract

The synovial tissue is an immunologically challenging environment where, under homeostatic conditions, highly specialized subsets of immune-regulatory macrophages and fibroblasts constantly prevent synovial inflammation in response to cartilage- and synovial fluid-derived danger signals that accumulate in response to mechanical stress. During inflammatory joint diseases, this immune-regulatory environment becomes perturbed and activated synovial fibroblasts and infiltrating immune cells start to contribute to synovial inflammation and joint destruction. This review summarizes our current understanding of the phenotypic and molecular characteristics of resident synovial macrophages and fibroblasts and highlights their crosstalk during joint homeostasis and joint inflammation, which is increasingly appreciated as vital to understand the molecular basis of prevalent inflammatory joint diseases such as rheumatoid arthritis.

## Introduction

### The Synovial Microenvironment

**FIGURE 1:**
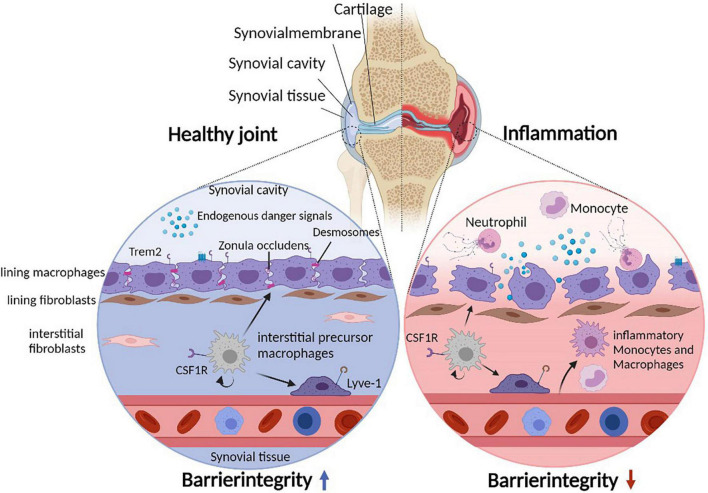
Cellular heterogeneity of the synovial microenvironment. Due to constant mechanical stress, the synovial cavity harbors endogenous danger signals. The synovial lining layer separates the synovial cavity from the tissue and consists of lining macrophages and fibroblasts. Lining macrophages express Trem2 and tight junctions (Zonula Occludens) as well as desmosomes, allowing maintenance and integrity of the synovial barrier. In addition, the subling interstitial tissue hosts various interstitial macrophage (and fibroblast) populations. During joint inflammation such as in patients suffering from rheumatoid arthritis, the lining integrity is disrupted and inflammatory immune cells infiltrate the tissue and the cavity. Under such inflammatory conditions, lining macrophages change their morphology to a phagocytic phenotype.

The synovial joint is a complex structure that connects distinct skeletal elements to allow locomotion. The corresponding articular bones are covered by articular cartilage and separated by synovial fluid that fills the spacing of the synovial cavity. A joint capsule supports the stability of the joint from the outside, whereas an inner synovial membrane physically encloses the synovial cavity separating its fluid from exterior joint structures (Figure 1). The synovial membrane itself consists of a lining layer that is enriched in macrophages (type A synoviocytes) and synovial fibroblasts (type B synoviocytes), while the sublining interstitial synovial tissue additionally harbors mast cells, adipocytes, blood vessels, lymphocytes, and heterogeneous populations of interstitial macrophages and fibroblasts (1, 2).

Apart from physically enclosing the synovial cavity, the synovial tissue fulfills several important physiological tasks that ensure proper joint function and which include the control and regulation of the composition of the synovial fluid and the consequent support of cartilage homeostasis.

During various forms of joint inflammation such as during rheumatoid arthritis (RA), psoriatic arthritis or also during degenerative joint diseases such as osteoarthritis (OA), the synovial membrane undergoes substantial changes in morphology and cellular composition and thereby becomes enlarged and inflamed. These inflammation-associated changes of the synovial tissue are best studied during different stages of RA, where the formation of a synovial pannus is a typical hallmark of joint pathology and characterized by synovial hyperplasia consisting of activated macrophages and fibroblasts as well as of infiltrating monocytes, granulocytes, lymphocytes, and monocyte-derived osteoclasts, which are considered to drive bone and cartilage destruction (2–4).

These observations have led to the perception and the concept that cells of the synovial tissue, and especially synovial macrophages and fibroblasts, critically contribute to joint inflammation and joint destruction during RA. Notably, however, emerging molecular data that have provided insights into the complex cellular heterogeneity of resident synovial cells and their diverse biologic functions indicate that the synovial membrane and in particular the various populations of resident synovial macrophages and fibroblasts exert differential and partially diverging pro- and anti-inflammatory functions that can both prevent and promote synovial inflammation (1, 3, 5–7).

Among the different resident synovial cell types, synovial fibroblasts and synovial macrophages have thus emerged as central regulators of synovial tissue homeostasis and factors determining onset and progression of inflammatory joint diseases such as RA. Our current review therefore focuses on the role of these two heterologous cell types with a particular emphasis on their role during the pathogenesis of RA. Notably, fibroblasts and macrophages closely associate and co-inhabit different microenvironmental niches of the synovial tissue, highlighting the importance of better understanding both the individual roles of these cellular subsets as well as their mutual interactions and influence. Insights into molecular pathways underlying their functional properties and crosstalk during homeostasis and inflammation is essential to understand the events that trigger and perpetuate inflammatory joint disease and will help to develop novel therapeutic concepts for the prevention and the treatment of prevalent chronic inflammatory musculoskeletal disorders such as RA and OA, which represent disabling diseases that do not only impose a significant health problem for millions of patients, but also an increasing socioeconomic burden for societies worldwide (8).

## The Golden Scale: Synovial Macrophage Heterogeneity in Health and Disease

The history of macrophage research started as early as 1883 when Ilja (Élie) Metchnikoff first described these cells and described phagocytosis as a central host defense mechanism, which substantially paved the way for our current understanding of the cellular base of innate immunity. Notably, Metchnikoff already anticipated the various functions of macrophages beyond immunity during the control of tissue homeostasis and turnover (9). Recently, new sequencing and imaging techniques have confirmed his early assumptions and allowed to shed more light on the plethora of macrophage-mediated functions as well as on their diverse developmental origin. Macrophages are now appreciated to play vital roles during host defense, the developmental establishment of organs and the maintenance of tissue homeostasis (10, 11). Despite their physiological relevance, however, these cells have been also implicated in the pathogenesis of various inflammatory and autoimmune diseases such as RA (7, 12, 13).

During various types of inflammation, the majority of macrophages derives from circulating blood monocytes that infiltrate the inflamed tissue and differentiate into newly recruited macrophages that display a pro-inflammatory gene expression pattern and consequently participate in host defense and the inflammatory response (14–17). During steady state, however, most tissue macrophage subsets throughout the body have a more diverse ontogeny and often do not directly derive from blood monocytes. These resident tissue macrophages mostly self-maintain their numbers independently of blood monocytes and are often established prenatally (16, 18–21). Classical examples of such monocyte-independent tissue resident macrophages are microglia in the brain (19, 22), Kupffer cells of the liver (23), and alveolar macrophages (24) in the lung. However, related populations of tissue resident macrophages can be as well found in other tissues such as the heart (25) or the bones (26). In contrast to their monocyte-derived counterparts in inflamed tissues, resident macrophages usually lack the expression of pro-inflammatory genes and usually display an anti-inflammatory phenotype. Another specific feature of such tissue resident macrophages is their close interconnection with stromal and parenchymal cells of their specific microenvironmental niche (27, 28). Thereby, they acquire distinct phenotypes that are reflected by tissue-specific transcriptional signatures and epigenetic profiles (29, 30). Especially the different (often tissue- and organ-specific) populations of fibroblasts interact and communicate with their corresponding counterparts on the side of tissue resident macrophages (3, 5, 27, 31). These functional modules consisting of tissue macrophages and fibroblasts seem to exert an important and mutual influence on each other and, together with other factors of the local microenvironment, shape the macrophage’s phenotypic and functional properties (27). Such tissue resident macrophages that are part of the organ-specific stromal scaffold then contribute to various organ-specific functions such as synaptic pruning in the CNS or metabolic adaption of the liver and adipose tissue (32–35). Apart from such organ-specific functions, however, resident macrophages throughout the body also share common properties. An important common denominator is their ability to rapidly and efficiently clear apoptotic cells and damage-derived danger signals allowing them to prevent spontaneous damage-induced inflammatory reactions in an otherwise healthy tissue microenvironment (28, 36, 37). The generally high cell turnover in barrier tissues such as the gut and skin seems to partially overextend the clearance capacity provided by the local population of tissue resident macrophages and therefore triggers the constant influx of additional monocyte-derived macrophages that help to maintain a necessary pool of tissue macrophages required to sustain tissue homeostasis (20, 22, 38–40).

### Macrophage Heterogeneity Within the Synovial Tissue

For decades, macrophages were considered main drivers of RA-associated joint pathology as they were found to be enriched in histopathological samples of RA patients where they contribute to synovial pannus formation (2, 4). Moreover, macrophages populating the inflamed synovial tissue of RA patients were shown to highly express proinflammatory cytokines such as TNF and IL-1β, provide matrix metalloproteinases (MMPs) and act as important source of reactive oxygen species thereby potentially contributing to inflammation, synovial tissue damage and cartilage degradation (41–45). More recently, however, a combination of complex fate mapping approaches, high resolution imaging, single-cell RNA sequencing (scRNA-seq) and computational analysis allowed uncovering a more complex role of synovial macrophages during synovial tissue homeostasis and RA, respectively. These insights revealed a high degree of developmental and functional heterogeneity of different synovial macrophage populations in the murine and human joint and showed that these cells exert diverse and partially opposing functions during joint inflammation (1, 5, 7, 46).

During steady-state, the synovial tissue hosts distinct populations of tissue resident macrophages that show close similarities and overlapping features in murine and human joints. One dominant population are synovial “lining macrophages” that are located at the top lining layer of the synovial membrane and are characterized by a combination of surface markers including Trem2 and CX_3_CR1 in mice as well as Trem2 and MerTK in humans. Another population of murine Lyve1^+^Relma^+^ (and human Lyve^+^MerTK^+^Folr2^high^) macrophages additionally populates the underlying interstitial synovial tissue. Such Lyve1^+^Relma^+^ macrophages most likely represent vessel-associated macrophages as their gene expression profile resembles perivascular macrophages in other organs such as the lung, heart, brain, and dermis (1, 47, 48). The murine interstitial synovial tissue additionally harbors a heterogeneous population of MHCII^+^CSF1R^+^ macrophages that resemble human MerTK^+^Folr2^+^ID2^+^ synovial macrophages. Onset of arthritis during experimental animal models and during human RA, in turn, is accompanied by the influx of additional populations of macrophages that show a typically pro-inflammatory gene expression pattern. These recruited macrophages secrete TNF and IL-1β, express chemokine receptors such as CCR2 in mice (1) and are MerTK^neg^CD48^high^S100A12^pos^ in human (5). During joint inflammation, these cells infiltrate both the interstitial synovial tissue and the synovial lining layer (1, 5, 7, 45, 46, 49).

### Ontogeny of Synovial Macrophages

Studies using parabiotic mice as well as genetic fate-mapping studies indicate that all three populations of resident synovial macrophages present during steady state (Trem2^+^CX_3_CR1^+^ lining macrophages, Lyve1^+^Relma^+^ perivascular macrophages and MHCII^+^CSF1R^+^ interstitial macrophages) are maintained independent of blood monocytes and suggest that these resident synovial macrophage subsets are established at a prenatal stage of development. These studies additionally indicate that MHCII^+^CSF1R^+^ interstitial macrophages are able to proliferate and represent a pool of “precursor macrophage” that constantly replenishes the pool of the other two synovial macrophage subsets of Trem2^+^CX_3_CR1^+^ lining macrophages and Lyve1^+^Relma^+^ perivascular macrophages that both show a higher degree of differentiation, but have lost their proliferative capacity (1). An important and currently unanswered question is the exact nature of factors that triggers the proliferation of MHCII^+^CSF1R^+^ interstitial macrophages as well as their subsequent differentiation of the more differentiated synovial macrophage subsets of Trem2^+^CX_3_CR1^+^ lining and Lyve1^+^Relma^+^ perivascular macrophages. Likely candidates include different micro-environmental factors such as small metabolites, components of the synovial fluid and/or the extracellular matrix as well as stromal-derived growth factors that are produced by synovial fibroblasts. Similar studies with arthritic mice show that CCR2- and IL1β-expressing inflammatory macrophages that become abundant in the inflamed synovial tissue represent a distinct population of macrophages that directly originate from blood monocytes, which are readily recruited upon onset of synovial inflammation (1).

### Resident Synovial Lining Macrophages Form an Anti-inflammatory Barrier

The Trem2-expressing subset of resident lining macrophages form a unique barrier-like structure between the fluid-filled synovial cavity and the interstitial synovial tissue in both mice and human (Figure 1). These lining macrophages are uniquely characterized by their ability to form tight junctions (Zonula Occludens) as well as desmosomes, express aquaporin channels and additionally show signs of an apical/basal polarization (1, 5). These findings indicate that they possess various features that are otherwise typical of epithelial cells in barrier tissues including the gut (50), which likely allow these macrophages to tightly enclose the fluid-filled synovial cavity and to simultaneously sense and regulate the molecular composition of the synovial fluid. Notably, however, synovial lining macrophages do possess high phagocytosis capacities, a feature that might be necessary to cope with a specific problem of the synovial microenvironment: the continuous presence of mechanical stress within the joint space (51). This mechanical stress results in constant and usually reversible cartilage damage as well as the degradation of integral components of the synovial fluid including hyaluronic acids and other glycosaminoglycans. This process results in the generation of various form of cellular debris and typical endogenous danger signals such as hyaluronan fragments that would potentially accumulate within the synovial fluid and subsequently induce synovial inflammation via activation of pattern recognition receptors such as Toll-Like Receptor (TLR) 2 and TLR 4 (52, 53), which are highly expressed in synovial lining macrophages and synovial fibroblasts (1, 54, 55). The microarchitecture of the synovial tissue and the absence of inflammation during steady state, however, suggests that lining macrophages constantly remove and clear such debris as well as the associated molecular danger signals in a non-inflammatory manner thereby preventing spontaneous joint inflammation. RNA sequencing indeed revealed that this macrophage subset lacks the expression of pro-inflammatory cytokines, but expresses a panel of immune-regulatory genes such as VSIG4 (V-Set and Immunoglobulin Domain Containing 4) and receptors that mediate efferocytosis including Axl, MerTK, Marco, and Tim4 allowing them an efficient non-inflammatory clearance of danger signals and apoptotic cell debris while maintaining an anti-inflammatory phenotype in an otherwise pro-inflammatory environment. These immune regulatory properties of synovial lining macrophages can be observed even upon onset of synovial inflammation during experimental arthritis and human RA (1, 5, 56). Synovial lining macrophages thereby appear to create an “immune-privileged microenvironment” and form an anti-inflammatory inside-outside barrier that normally prevents both a danger signal-induced spontaneous inflammation of the synovial tissue in response to mechanical stress as well as an inflammation-induced cell trafficking into the inner joint structures. On a functional level, the synovial barrier is therefore partially comparable both to the gut barrier that prevents spreading of danger signals into the surrounding intestinal tissue as well as to the blood-brain barrier that limits immune cell trafficking into the CNS.

Onset of synovial inflammation during murine arthritis models and human RA is associated with a break-down of this protective epithelial-like macrophage structure (1, 5). During arthritis, lining macrophages substantially change their morphology and lose their cell-cell contacts resulting in an increased permeability of the synovial membrane. Upon onset of experimental arthritis and during human RA, there is also a relative decrease in the number of Trem2-expressing resident lining macrophages and an increase in infiltrating CCR2- and IL-1β-expressing monocyte-derived macrophages, which is reversed again upon clinical disease remission where the anti-inflammatory tissue resident macrophage populations dominate again and the synovial barrier reforms (1, 5).

In line with an important anti-inflammatory role of the Trem2^+^ lining macrophage subset, selective depletion of murine synovial lining macrophages (1) as well as global deletion of resident synovial macrophages (46) resulted in early onset and exacerbation of arthritis in mice, emphasizing their crucial role in the maintenance of joint homeostasis and the prevention of synovial inflammation. Data from human RA patients confirm an anti-inflammatory role of resident synovial macrophages and show that these cells are potent producers of anti-inflammatory pro-resolving lipid mediators and simultaneously promote a repair response of synovial fibroblasts (5). The breakdown of the synovial macrophage barrier during RA thus seems to be both a cause and a consequence of inflammation and might be part of a vicious cycle responsible for the chronicity of synovial inflammation, which is typically observed during RA and related inflammatory joint diseases.

### Immune-Regulatory Perivascular Macrophages Dominate the Interstitial Synovial Tissue During Steady State

The sublining interstitial tissue comprises fibroblasts, nerves, blood vessels as well as additional populations of phenotypically distinct synovial macrophages (3, 7). A dominant population within the murine synovial interstitial tissue are Lyve1^+^Relma^+^ macrophages. Computational and experimental approaches show that related perivascular macrophages are conserved across different organs (e.g., heart, lung, dermis, and brain) (47, 48, 57, 58). All these Lyve1^+^Relma^+^ macrophage subsets express high levels of a distinct set of chemokines such as CCL-24 and CCL-12, which are important for regulating leukocyte trafficking. Interestingly, these cells are also enriched in anti-inflammatory cytokines including IL-10 and TGF-β, and additionally express markers typical of alternatively activated macrophages such as mannose-receptor 1 (CD206) and the receptor for the hemoglobin-haptoglobin complex (CD163). These latter phenotypic features indicate additional immune-regulatory properties of this macrophage subset. Indeed, they seem to regulate blood vessel permeability, control leukocyte migration and might participate in the resolution of inflammation (47, 57). Specific depletion of such Lyve1^+^ Relma^+^ macrophages resulted in excessive fibrosis in the lung as well as arterial stiffness underscoring a predominant anti-inflammatory and pro-resolving role of these cells (47, 57). Their exact role during synovial tissue homeostasis and synovial inflammation, however, remains to be addressed in more detail.

### Monocyte-Derived Macrophages Infiltrate the Inflamed Synovial Tissue

A hallmark of arthritis is the infiltration of immune cells including monocytes and monocyte-derived macrophages. Recent advances in the field of single-cell RNA sequencing together with insights from fate mapping studies, show that, upon onset of arthritis, blood-derived CCR2^+^ monocytes start to infiltrate the synovial tissue and give rise to different populations of monocyte-derived macrophages that are characterized by a pro-inflammatory activation profile (including high expression levels of IL-1β). On a phenotypic level, these monocyte-derived macrophages thus substantially differ from the described subsets of specialized resident synovial macrophages that dominate during steady state and that display an immune regulatory and anti-inflammatory activation profile. In accordance with the pro-inflammatory role of monocytes and monocyte-derived macrophages, depletion of these cells diminished joint pathology and accelerated the resolution of inflammation during experimental arthritis models (1, 46). In human synovial tissue, three populations of CD206^neg^MerTK^neg^ macrophages, which resemble infiltrating CCR2^+^ monocytes and pro-inflammatory monocyte-derived macrophages in mice, seem to contribute to synovial inflammation as well as to RA-associated bone destruction (1, 5, 7).

## More Than a Passive Scaffold: Synovial Fibroblasts Shape Their Environment

Fibroblasts were first described as a distinct cell type in the late nineteenth century by Rudolph Virchow and Mathias Duval as “spindle shaped cells of the connective tissue,” referring to their morphological shape. The term fibroblast was introduced later to describe cells that were observed to produce new connective tissue in healing organ structures (59–61). As structural cells, fibroblasts are ubiquitously present throughout mammalian tissues. A shared function of all fibroblasts is the ability to synthesize and deposit major components of the extracellular matrix (ECM), which includes proteoglycans, collagens, fibronectin or elastin. Apart from the creation of ECM, however, these cells exert versatile roles during tissue homeostasis, inflammation and repair, where the fibroblast acts as a jack of all trades that shapes its tissue specific microenvironment. Production of lysyl oxidase and lysyl hydroxylases allow fibroblasts to modulate cross-linking between collagens and elastin thereby fine-tuning the physical properties of the ECM (62). Fibroblasts also express MMPs as well as MMP inhibitors allowing them to remodel the ECM depending on the current necessity. Fibroblasts are important participators in repair processes where they do not only contribute to new ECM, but simultaneously serve as progenitor cells for other mesenchymal cell types, such as adipocytes, chondrocytes or bone-forming osteoblasts (62, 63). Notably, fibroblasts closely interact with tissue macrophages and other cells of the innate and adaptive immune response (64). Here, they act as important sentinel cells that sense and recognize danger signals derived from pathogens or damaged and dying cells. In response to damage and danger, fibroblasts then activate pro-inflammatory signaling pathways in order to support the recruitment and activation of polymorphonuclear neutrophils, monocytes and lymphocytes (65–67).

Novel scRNA-seq-based studies that studied fibroblasts across different organs show that, despite such common features, fibroblasts also show a substantial heterogeneity that might enable these cells the execution of tissue—specific functions (68). Studies on fibroblasts in murine heart, skeletal muscle, intestine and bladder have shown a high degree of diversity within their transcriptome where only 20% of fibroblast genes were shared across these four organs (69). In line with these findings, another study showed that a fibroblast’s scRNA-seq profile more closely resemble that of other structural cells within the respective organ than that of fibroblasts within other organs (70). Locally imprinted transcriptional profiles thus likely mirror the fibroblast’s unique functionalities within its specific tissue environment. In addition to this heterogeneity on a global scale, fibroblast can also be differentiated into functionally distinct subtypes within a single tissue, highlighting that their identity is shaped by specific and local microenvironmental niches they inhabit (71). Even within the limbs, differing transcriptional profiles have been detected in the dermal mesenchyme of the torso, of the fingers and of toes (72, 73). This positional identity can be retained *in vitro* indicating these cells are permanently primed by local niche-specific factors (74).

### Synovial Fibroblast Niche

Fibroblasts are abundant cells within synovial membrane. Previous data indicate that these cells contribute to the pathogenesis of inflammatory joint disease at different levels. Activation of synovial fibroblasts during RA seems to takes place at a very early disease stage, where activation of TLR2, 3 and 4, which have been found to be expressed on synovial fibroblasts in RA patients, might act as important inflammatory triggers of these cells (75, 76). The activation of synovial fibroblasts seems to result in extensive alterations of the synovial tissue microenvironment and remodeling of the ECM through the secretion of matrix-degrading enzymes, the production of proangiogenic factors and the expression of pro-inflammatory cytokines and chemokines (83, 104, 107, 108, 110, 112). Fibroblasts contribute to synovial inflammation via the production of IL-6, and chemokines like IL-8, CCL2, CCL3, and CCL5 (105, 106, 109, 111). Especially fibroblast—derived IL-6 has been implicated in the pathogenesis of RA and meanwhile emerged as a major therapeutic target in the treatment of RA patients (77, 78). Fibroblast-derived RANKL, in turn, has been implicated in the enhanced differentiation of osteoclasts as well as the osteoclast-mediated destruction of periarticular bone observed in RA patients and thus presents another therapeutic target (79–81).

Once activated, synovial fibroblasts express increased levels of adhesion molecules including different integrins and cadherins, resulting in attachment and invasion of articular cartilage. Specifically, cadherin-11 was found to be pivotal for the invasive potential of inflammatory synovial fibroblasts and blockage of the cadherin-11 accordingly ameliorated cartilage destruction in experimental arthritis model (82, 83). Furthermore, synovial fibroblasts secrete growth stimulating factors such as B-cell activating factor (BAFF) and a proliferation—inducing ligand (APRIL) that promote survival of B cells and might contribute to the establishment and maintenance of tertiary lymphatic structures within the synovial tissue of RA patients (65, 84).

Within the synovial tissue, two functionally and phenotypically distinct populations of fibroblasts emerged from recent studies that built on scRNAseq-based analyses. These studies could differentiate transcriptionally distinct lining fibroblasts, which co-localize with the macrophage lining layer of the synovial membrane, from sublining fibroblasts that are situated deeper within the synovial tissue (1, 3). Lining and sublining fibroblasts, however, do not appear to represent two completely separated fibroblast entities, but rather terminal points of a continuous differentiation spectrum. This is evident from the varying expression of various fibroblast markers including THY1 and proteoglycan 4 (PRG4). Lining synovial fibroblasts express PRG4, but hardly any THY1, whereas sublining synovial fibroblast express THY1 but no PRG4. According to the expression of these markers, most synovial fibroblasts actually fall in between these two poles (3, 85, 86). The placement along this THY1:PRG4 gradient seems to be a result of the positional identity of these cells and dependent on the spatial distance from endothelial cells and endothelial cell-derived NOTCH3 signaling (3, 6). During arthritis, both the pool of lining and sublining fibroblasts is disturbed and can expand accordingly. The different positional features of synovial fibroblasts, however, are paralleled by a distinct behavior within the inflamed synovial tissue.

THY1 + sublining sublining fibroblasts were shown to primarily promote inflammation in response to increased endothelial-edrived Notch3 signaling with minimal effect on bone and cartilage damage during arthritis (3, 6). In active RA, Notch3 and Notch target genes are accordingly upregulated in synovial sublining fibroblasts, explaining the expansive inflammatory behavior of these cells. THY1-negative lining fibroblasts, in turn, seem to selectively mediate bone and cartilage damage with little effect on inflammation. Lining layer fibroblasts indeed induce osteoclastogenesis and subsequent cartilage and bone erosion mainly through the secretion of RANKL (3, 79, 81, 87).

## Crosstalk Between Synovial Fibroblasts and Macrophages

Understanding how heterologous cell types such as synovial fibroblasts and synovial macrophages communicate and interact with each other might be a key to understand disease mechanisms and identify novel therapeutic approaches in inflammatory joint disease. A close physical interaction and signaling loops between fibroblast-like cells and various tissue-specific macrophage subsets can be observed throughout the body where, e.g., osteoblasts and osteoclasts or astrocytes and microglia closely interact and exert a mutual influence on each other (88, 89). Local factors throughout the body shape the tissue-specific phenotypes of both macrophages and fibroblasts during steady state and inflammation. These include changes in the local concentration of various metabolites such as retinoic acid or lactate as well as variations in oxygen tension that impact both on the local pool of macrophages and fibroblasts (90, 91). Also, the presence of unique factors that shape the specific microenvironmental niche such as surfactant in the lung, bacteria in the gut or synovial fluid within the joints likely imprint on the phenotypic features of both cell types. Both macrophages and fibroblasts, in turn, also influence their environment and remodel the ECM, produce and resorb surfactant and synovial fluid or imprint on the local metabolic tissue signature (92, 93).

**FIGURE 2:**
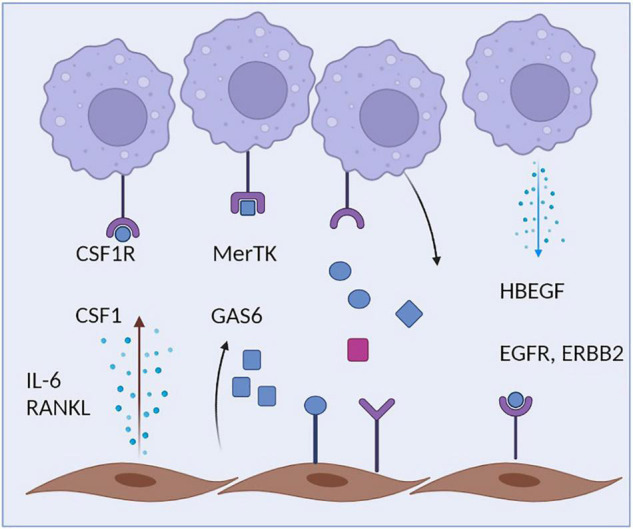
Crosstalk of macrophages and fibroblasts. Synovial macrophages are capable of sensing multiple fibroblast-derived factors including Csf1 and Gas6. During inflammation, Synovial fibroblasts additionally secrete IL-6, which amplifies the inflammatory response of both macrophages and fibroblasts. Synovial fibroblasts, in turn, rely on macrophage-derived HBEGF.

The close physical association of fibroblasts and macrophages is also reflected by an intense cellular communication between both cell types. Fibroblasts are probably the most relevant source of CSF1 within the microenvironment of tissue macrophages (Figure 2). Fibroblast-derived CSF1, in turn, binds to the CSF1R on macrophages promoting the survival, proliferation and maintenance of the local macrophage pool (31, 94). Mice lacking CSF1 also lack most macrophage subsets and deletion of CSF1 in fibroblasts results in related phenotypes (95). Within many tissues, there seems to be a symbiotic relationship between macrophages and fibroblasts that does not only involve CSF1 production by fibroblasts, but also the expression of PDGF by macrophages, a cytokine that exerts a strong impact on the maintenance of tissue fibroblasts and their proliferative capacity (27). These insights point toward a complex two cell circuit by which these cells reciprocally interact and sustain their numbers and shape their phenotypes within the tissue context (31). The specific requirements and factors that allow the interaction and communication of synovial macrophages and fibroblasts within the specific niches of the synovial lining and synovial sublining interstitial tissue and their potential for targeted therapy remain to be identified. This homeostatic equilibrium and balanced interaction might become disturbed during various disease states where macrophages and/or fibroblast activity is altered and consequently affects the other cell type. Excessive production of cytokines such as IL-6 and of chemokines such as CCL2 by fibroblasts or increased secretion of TGFbeta and PDGF by macrophages might be the consequence thereby contributing to excessive inflammation and/or a fibrotic reaction Such pathologic circuits have been described to contribute to diseases such as lung fibrosis, but might well be involved in synovial inflammation and synovial pannus formation during RA as well (90, 96). Cellular communication between macrophages and fibroblasts seem to partially amplify both a pro-fibrotic response in fibroblasts and a pro-inflammatory response in macrophages, thereby driving a vicious circle of TGFbeta production by macrophages and IL-6 production by fibroblasts (97, 98). Activated synovial fibroblasts were recently described to have the capacity to influence the metabolic rewiring of synovial macrophages and skew their metabolic profile toward an upregulation of glycolysis and mitochondrial respiration as well as an increase in uptake of glucose and glutamine, a process that may exert a long lasting influence on the inflammatory behavioral pattern of these ells (99). During synovial inflammation, production of HBEGF (heparin binding EGF-like growth factor) by inflammatory macrophages seem to promote the invasive behavior of resident synovial fibroblasts and the consequent destruction of articular cartilage (100). These findings are in accordance with the observation that human CD206^neg^MerTK^neg^ (monocyte-derived) synovial macrophages promote an inflammatory phenotype in synovial fibroblasts when both cells are co-cultured. CD206^pos^MerTK^pos^ macrophages, which include the resident subsets of Trem2^+^ lining and Lyve1^+^ interstitial macrophages, in contrast, trigger a repair response in synovial fibroblasts. The consequently emerging anti-inflammatory homeostatic response includes the production of GAS6 by synovial fibroblasts, which in turn induces a pro-resolving and immune-regulatory phenotype in resident synovial macrophages that express the GAS6 receptor MerTK. GAS6 deletion in fibroblasts accordingly provokes a pro-inflammatory macrophage response (5).

During the treatment of chronic inflammatory diseases such as RA, our current therapeutic armory that is increasingly build on monoclonal antibodies, is already able to successfully target both fibroblast-derived cytokines such as IL-6 as well as macrophage-derived cytokines such as TNF and thus interfere with the inflammatory crosstalk between both cell types (4). Newer small molecular therapeutics such as JAK-inhibitors seem to target pro-inflammatory signaling in both types of cells, which might explain the relatively broad and superior anti-inflammatory and anti-fibrotic properties of this class of drugs (101). Attractive emerging targets include inhibitors of cycline-dependent kinases that suppress proliferation of activated synovial fibroblasts (102). Future therapeutic strategies will likely benefit from the synergistic action of such different classes of drugs that allow a combinatorial targeting of different synovial cell types thereby limiting their crosstalk and interrupting the vicious cycle of chronic synovial inflammation in diseases such as RA (103).

## Conclusion

Distinct resident synovial macrophage and fibroblast populations that populate the synovial lining layer and the synovial interstitial tissue closely interact with each other and shape specific niches within the synovial microenvironment. Resident synovial Trem2-expressing lining macrophages associate with a phenotypically distinct population of THY1-negative fibroblasts and form a dense synovial lining layer. Both cell types contribute to the composition and turnover of the synovial fluid and simultaneously prevent spontaneous synovial inflammation by forming an anti-inflammatory barrier interface between the synovial fluid and synovial tissue that facilitates the constant clearance of synovial fluid-derived danger signals that accumulate due to mechanical stress. Upon onset of inflammation, this protective barrier breaches allowing newly recruited pro-inflammatory monocytes and monocyte-derived macrophages the access to inner joint structure and promoting a pro-inflammatory program in the population of THY1-negative fibroblasts that co-inhabit the synovial lining layer and consequently initiates joint destruction. Synovial inflammation also results in the activation of THY-expressing sublining fibroblasts that depend on endothelial-derived Notch3 and further promote synovial inflammation. The role of perivascular macrophages during arthritis is less clear as they might be theoretically involved both in the initiation and resolution of inflammation.

Future research needs to identify the local factors and interaction networks that shape the identity of these various cellular subsets and identify the causes and consequences during a complex series of events that promote onset and progression of synovial inflammation during RA and related disorders.

## Author Contributions

All authors contributed to the current manuscript and approved submission.

## Conflict of Interest

The authors declare that the research was conducted in the absence of any commercial or financial relationships that could be construed as a potential conflict of interest.

## Publisher’s Note

All claims expressed in this article are solely those of the authors and do not necessarily represent those of their affiliated organizations, or those of the publisher, the editors and the reviewers. Any product that may be evaluated in this article, or claim that may be made by its manufacturer, is not guaranteed or endorsed by the publisher.
